# A new and efficient enrichment method for metagenomic sequencing of Monkeypox virus

**DOI:** 10.1186/s12864-023-09114-w

**Published:** 2023-01-17

**Authors:** Pablo Aja-Macaya, Soraya Rumbo-Feal, Margarita Poza, Angelina Cañizares, Juan A. Vallejo, Germán Bou

**Affiliations:** 1 Microbiology Research Group, Biomedical Research Institute of A Coruña (INIBIC) - University Hospital of A Coruña (CHUAC) - CIBER of Infectious Diseases (CIBERINFEC), Servicio de Microbiología, 3ª planta, Edificio Sur, Hospital Universitario, As Xubias, 15006 A Coruña, Spain; 2 University of A Coruña (UDC) - Center for Advanced Research (CICA), Facultad de Ciencias, Campus Zapateira, 15008 A Coruña, Spain

**Keywords:** Human monkeypox (hMPX), *Monkeypox virus *(MPXV), Genome sequencing, Viral surveillance, Metagenomics, Host DNA depletion

## Abstract

**Background:**

The methodology described in previous literature for *Monkeypox virus* (MPXV) sequencing shows low efficiency when using metagenomic approaches. The aim of the present study was to evaluate a new fine-tuned method for extraction and enrichment of genomic MPXV DNA using clinical samples and to compare it to a non-enrichment metagenomic approach.

**Results:**

A new procedure that allows sample enrichment in MPXV DNA, avoiding wasting the sequencing capacity in human DNA, was designed. This procedure consisted of host DNA depletion using a saponin/NaCl combination treatment and DNase, together with high g-force centrifugations. After typical quality control, samples using the enrichment method contained around 96% of reads not classified as human DNA, while the non-enrichment protocol showed around 5-10%. When reads not belonging to *Orthopoxvirus* were removed, enriched samples kept about 50% of the original read counts, while non-enriched ones kept only 2-7%.

**Conclusions:**

Results showed a very significant improvement in sequencing efficiency, increasing the number of reads belonging to MPXV, the depth of coverage and the trustworthiness of the consensus sequences. This, in turn, allows for more samples to be included in a single cartridge, reducing costs and time to diagnosis, which can be very important factors when dealing with a contagious disease.

**Supplementary Information:**

The online version contains supplementary material available at 10.1186/s12864-023-09114-w.

## Background

Human monkeypox (hMPX) is a zoonosis disease originated in the jungles of Central and West Africa. This infectious disease was discovered in 1958 in two different monkey research colonies belonging to a Danish research institute [1]. It was described for the first time in a child in the Democratic Republic of the Congo in 1970. Later in the 1970s, forty seven cases of human monkeypox occurred in Central and West African Countries (Zaire, Nigeria, Liberia, Sierra Leone and Ivory Coast) [2]. In these areas the outbreaks of hMPX were reported in remote populations that depend on hunting and consume bushmeat [3]. Both rodents and monkeys can infect humans, however, it is not yet known which is the original reservoir of this disease [4].

The Global Commission for the Certification of Smallpox Eradication designated MPXV as the most important *Orthopoxvirus* infecting humans in the post-smallpox eradication era (from 1980). They recommended a surveillance program on MPXV and the study of its epidemiology and ecology [5].

Although the causes are unknown, since 1980 hMPX cases have gradually increased in Central Africa and more recently in West Africa [3]. In addition to this gradual increase in Africa, prior to 2022, hMPX cases outside of Africa were related to international travels or to animals imported from West and Central African countries [6]. However, from 2022, outbreaks with local transmission were established in multiple countries and continents [7]. Despite this increase, there is a lack of knowledge about hMPX emergence, epidemiology and ecology.

MPXV infects humans through contact with other infected humans and animals or with contaminated material. MPXV enters the body through broken skin, the respiratory tract or the mucous membranes. Before the 2022 outbreaks, animals were the main transmission route for hMPX. This could occur by bite or scratch, bushmeat preparation, direct contact with body fluids, or lesions from an infected animal or contaminated material. However, 2022 outbreaks in different countries and continents showed that the main transmission route was human to human. This human to human transmission occurs by respiratory droplets, through contact with bodily fluids from infected people or with contaminated objects [7].

MPXV is an enveloped double-stranded DNA virus. This virus belongs to the *Orthopoxvirus* genus of the *Poxviridae* family and has a genome size of approximately 197 Kb. MPXV shares its genus with 11 species that affect different animals, such as the variola virus, which are historically important viruses [7]. Two clades of MPXV are currently distinguished by genomic sequencing: the Central African and the West African. The Central African clade causes more severe disease and mortality [4].

MPXV and in general Poxviruses have excellent resistance to desiccation and wide pH tolerance compared with other enveloped viruses. These characteristics make the viral particles more stable in the environment. Materials from infected people or fomites could have infectious capacity during months or years. However, these viruses are sensitive to disinfectants, although less than others enveloped viruses [7].

The aim of the present study was to evaluate and compare a metagenomic sequencing approach of MPXV that uses a regular DNA extraction (non-enrichment metagenomic approach) with a new MPXV DNA enrichment methodology proposed using clinical samples. The methodology previously described in the literature [8–10] showed a large waste of sequencing resources. For example, Cohen-Gihon et al. [8] obtained a total of 2 M sequences from MPXV sequencing, and only 48 K sequences belonged to MPXV (1.8% of the total reads). Fuchs et al. [9] obtained 9 M reads from MPXV sequencing, and only 265 K reads belonged to MPXV (3% of the total reads). Israeli et al. [10] obtained 16.3 M reads from MPXV sequencing, and only 1 M reads belonged to MPXV (6.5% of all reads). Isidro et al. [11] obtained around  80 million total reads *per* sample using a NextSeq 2000 (Illumina) device, but only 4% of the reads belonged to MPXV. Overall, data indicates that over 90% of the sequencing effort was wasted.

In this study, efforts were focused on improving the performance of the MPXV sequencing processes in order to avoid wasting most of the reads on the human host. For this purpose, a saponin-based enrichment method was designed. The use of the nonionic surfactant saponin followed by a DNase treatment has shown to be highly efficient in the depletion of human DNA without affecting viral DNA [12, 13]. Thus, a novel protocol was developed for enrichment of MPXV DNA, in order to optimize the sequencing procedure, improving the coverage and the trustworthiness of the observed mutations.

## Results

A procedure that allows sample enrichment in MPXV DNA was designed, avoiding wasting the sequencing capacity in human DNA. This procedure consisted of host DNA depletion using a saponin/NaCl combination treatment and DNase. Prior to this, a soft centrifugation that allowed the removal of big particles and part of the eukaryotic cells was used. After human DNA depletion, it is crucial to remove saponin, NaCl and DNase to generate a library for sequencing. Samples were centrifuged at 35000 *g* and the MPXV particles washed three times using PBS. MPXV belongs to the *Poxviridae* family, characterized by being the most complex and largest viral family. This large size allowed their easy centrifugation at 35000* g *[14].

MPXV samples used in this protocol are listed in Table S1 and were collected from swabs of dermic vesicles. Two of the samples, MP01 and MP03 (anonymized identifiers), were processed using either the MPXV enrichment protocol proposed in this work (MP01CHUAC, MP03CHUAC) or the non-enrichment method (MP01bCHUAC, MP03bCHUAC). The rest of samples were directly subjected to the MPXV enrichment protocol. Sample groups were sequenced in three different runs (Table S1).

**Fig. 1:**
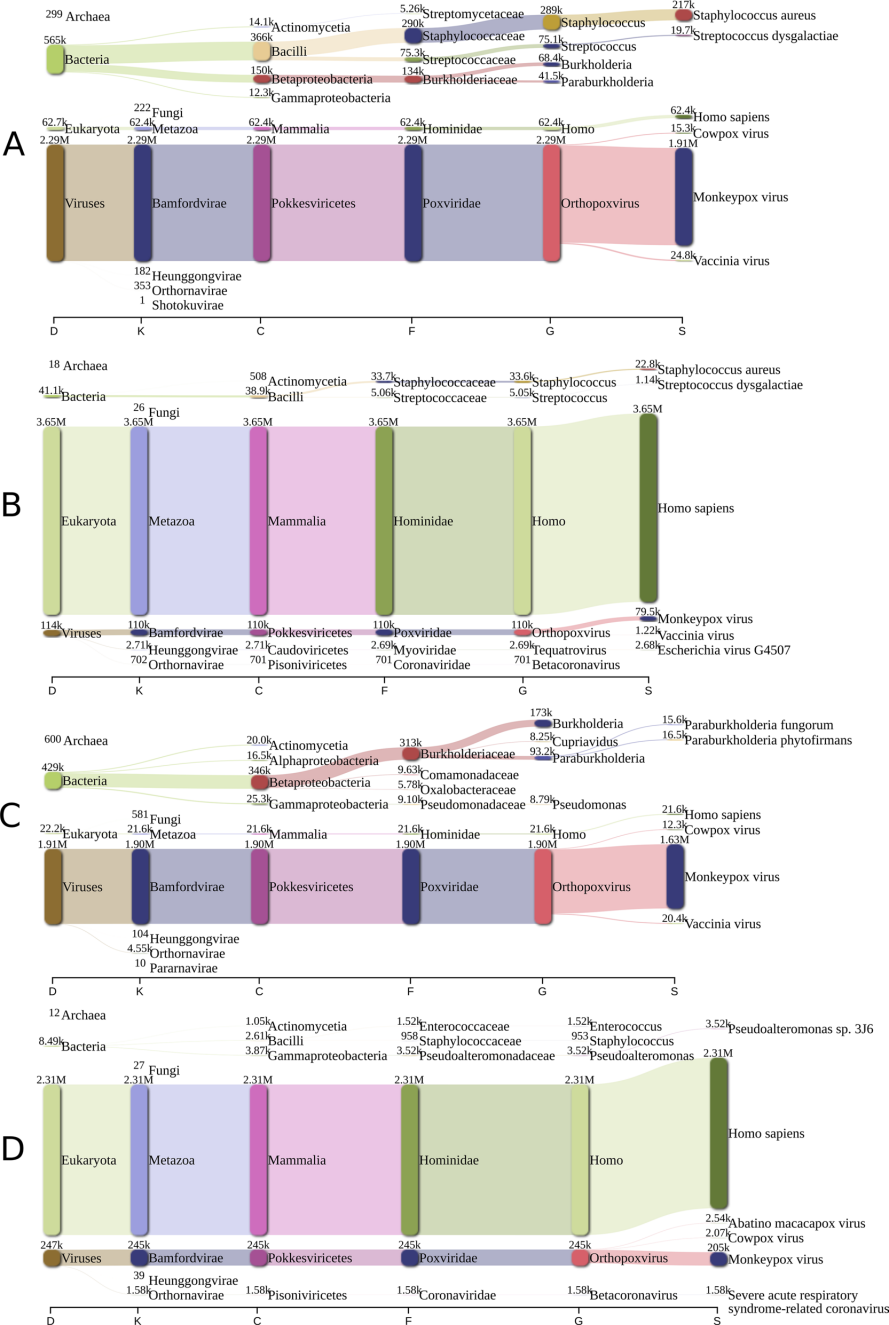
Raw read classification: These plots represent the classification Kraken2 has performed on the original reads (those without any filtering or quality control) of paired samples (**A**: MP01CHUAC (enrichment method), **B**: MP01bCHUAC (non-enrichment method), **C**: MP03CHUAC (enrichment method), **D**: MP03bCHUAC (non-enrichment method)) visualized using Pavian. The read counts are presented in pairs and the maximum taxa *per* level is 6. D: domain; K: kingdom; C: class; F: family; G: genus; S: species

Preliminary Kraken2 reports using the original reads (no filters or quality control) of paired samples (those that were tested with both methods), can be visualized in Fig. 1, showing a clear difference between samples. In MP01CHUAC and MP03CHUAC there were almost no reads classified as host contamination, whereas in MP01bCHUAC and MP03bCHUAC most of the reads belonged to human DNA.

**Fig. 2:**
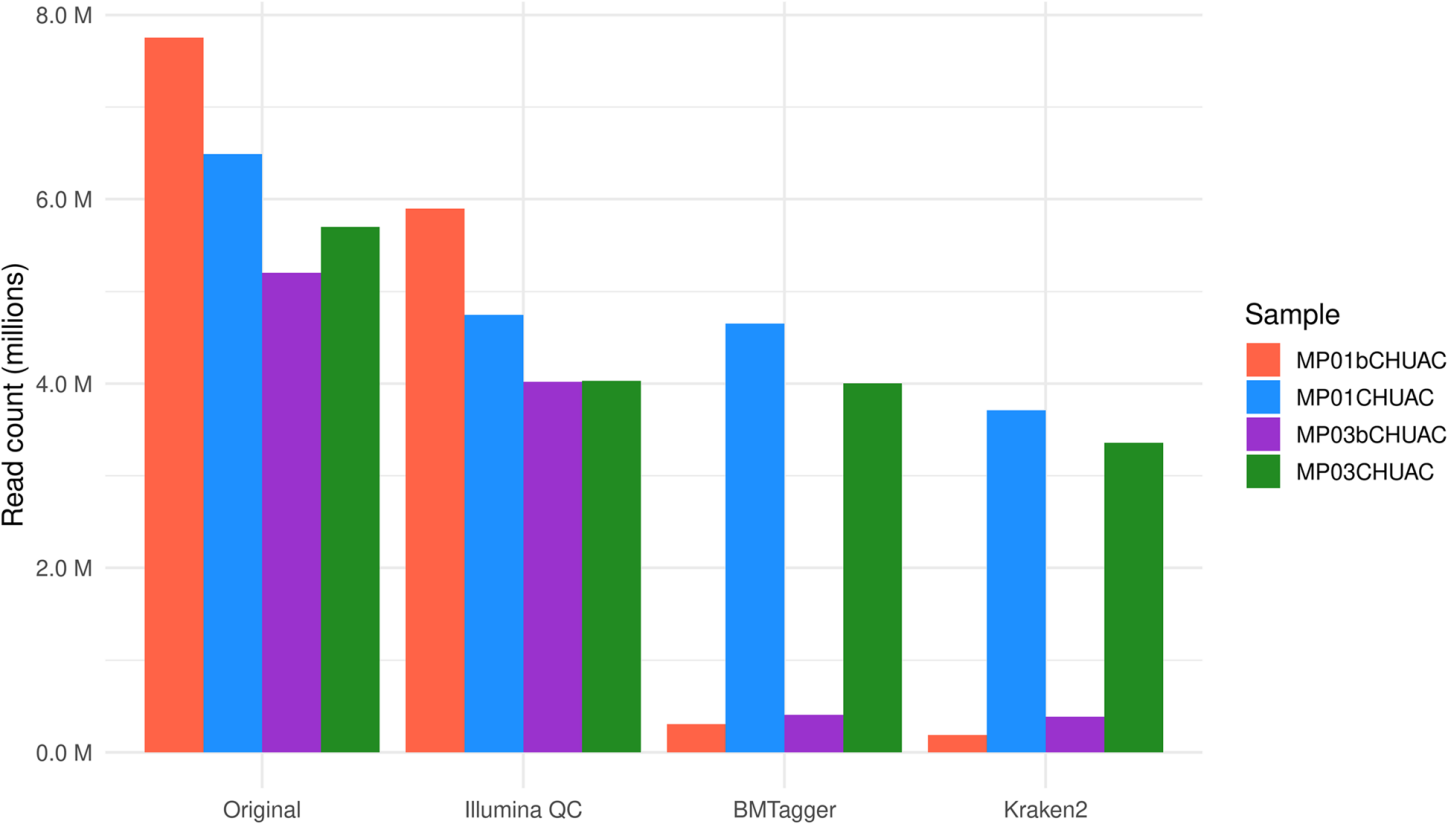
Read counts evolution across quality controls. The number of reads (counting both forward and reverse) is shown for each paired sample across various quality control steps. These steps are shown in order of execution, from left to right. Original: raw reads; Illumina QC: typical illumina quality control; BMTagger: removal of human reads; Kraken2: Removal of anything not belonging to the *Orthopoxvirus* genus. MP01CHUAC and MP03CHUAC followed the enrichment method whereas MP01bCHUAC and MP03bCHUAC followed the non-enrichment method

In Fig. 2, total read counts for each paired sample (including both forward and reverse reads) are presented for each quality control step. A significant change can be observed in the third step, BMTagger, where reads are classified into human or not and removed if they belong to the host. While read counts for MP01CHUAC and MP03CHUAC decreased from 4.75 M and 4.03 M reads to 4.65 M and 4.00 M, respectively (reduction of  1-2%), the read counts for MP01bCHUAC and MP03bCHUAC went from 5.9 M and 4.02 M to 0.3 M and 0.4 M, respectively (reduction of  90-95%). In the fourth step, Kraken2, where anything not classified as *Orthopoxvirus* is discarded, the differences were less drastic, with MP01CHUAC and MP03CHUAC having a reduction of  30%, while MP01bCHUAC and MP03bCHUAC showed a reduction of 41% and 8%, respectively. When comparing the original read count with the final quality controlled reads, a reduction of about 50% was observed for MP01CHUAC and MP03CHUAC, whereas for MP01bCHUAC and MP03bCHUAC it was around 93-98%. The exact counts for each step for paired samples and the rest of samples are available in Table 1. When taking into account the rest of samples, the median lost reads percentage for the enriched libraries was 48.26±9.93% ($n=10$), whereas for non-enriched ones it was 95.25±3.45% ($n=2$).

The alignment statistics of the remaining reads against the reference genome “MPXV_USA_2022_MA001” (ON563414.3) are presented in Table 2, where MP01-CHUAC and MP03CHUAC had a median depth of 1500-1800, whereas MP01b-CHUAC and MP03bCHUAC had around 80-100. Additionally, the rest of non paired samples sequenced in run C (Table 2) had a median depth of 296.5±137 ($n=8$).

**Fig. 3:**
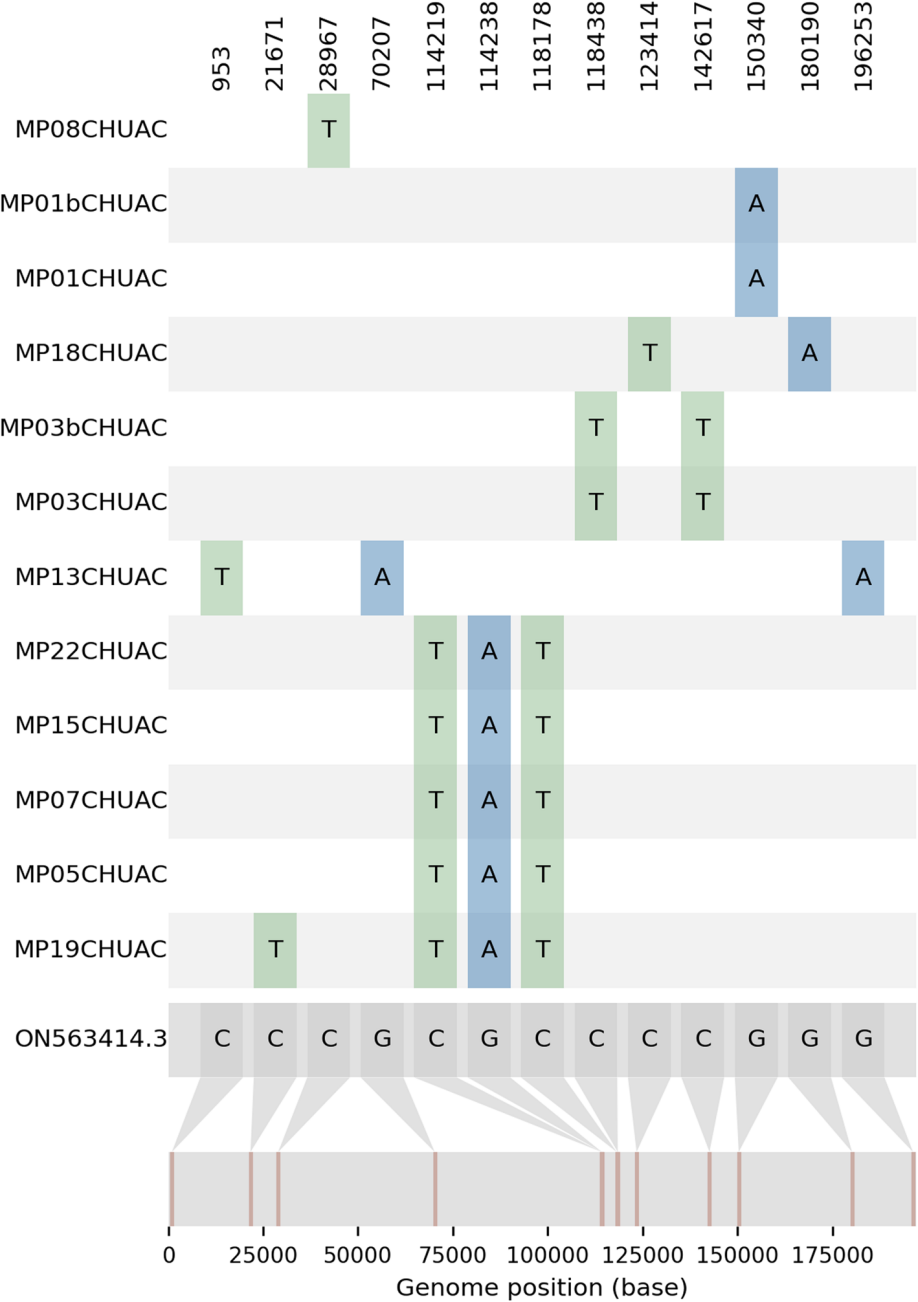
Substitutions detected in the MPXV used in this study: Single nucleotide variants (SNVs) for the samples used in this study are shown, comparing the mutations in each sample to the reference genome ON563414.3. MP01bCHUAC and MP03bCHUAC followed a non-enrichment method, the rest of samples followed the enrichment method

All samples were able to produce a good quality consensus sequence belonging to lineage MPXV B.1 using an alignment-based consensus approach. Their SNVs (single nucleotide variants) against the reference genome “MPXV_USA_2022_MA001” (ON563414.3) are shown in Fig. 3. In terms of amino acid substitutions, MP01CHUAC had none, MP03CHUAC had two (OPG136:M389I, OPG163:H136Y), MP08CHUAC had one (OPG045:D105N), MP13CHUAC had two (OPG001:E214K, OPG089:E109K), MP18CHUAC had two (OPG144:C179Y, OPG208:G316R) and a very defined group formed by MP05CHUAC, MP07CHUAC, MP15CHUAC, MP19CHUAC and MP22CHUAC had two amino acid substitutions (OPG133:D499N, OPG136:R476Q). However, this approach may show problems in highly repetitive regions, such as the characteristic inverted terminal repeats (ITRs), and in some hot-spots containing very large and highly variable insertions. Due to this, a *de novo* assembly approach was also tested, which resulted in the same mutations and structure, but differing in the length of some insertions.

**Fig. 4:**
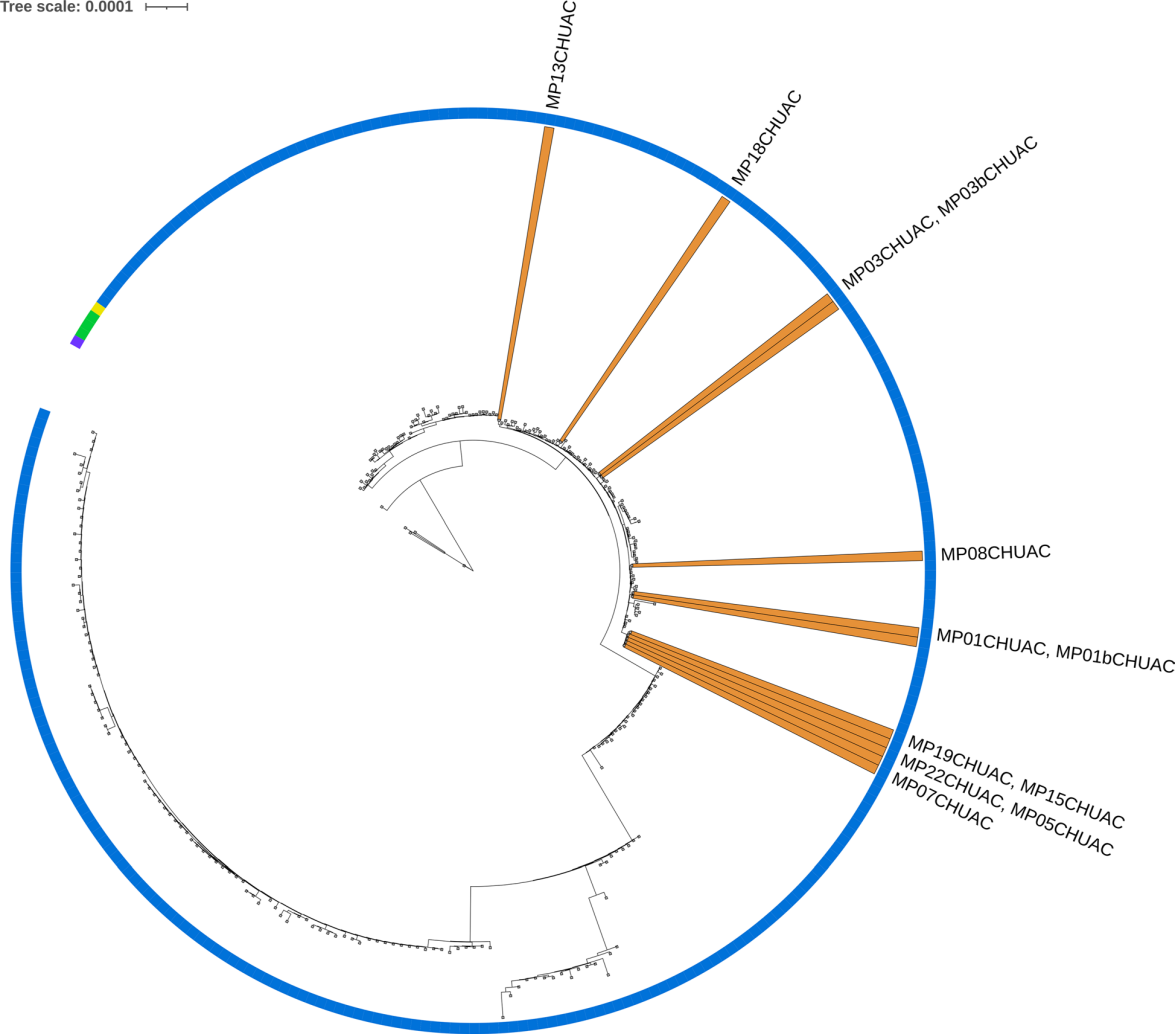
Phylogenomic tree. Phylogenomic analysis of the present study’s samples, comparing them to all complete MPXV genomes available in GenBank up to 2022-07-18 (275 genomes from taxid 10244). A color strip indicates each sample’s lineage (A: purple, A.1.1: yellow, A.2: green, B.1: blue), and orange areas highlight the study’s samples. MP01bCHUAC and MP03bCHUAC followed a non-enrichment method, the rest of samples followed the enrichment method

A phylogenomic analysis (Fig. 4) was also made to study the relatedness of the samples to all 275 complete MPXV genomes from taxid 10244 available in GenBank up to 2022-07-18 (Table S2).

## Discussion

The objective of this study was to evaluate and compare a MPXV metagenomic sequencing method using a regular DNA extraction (non-enrichment approach) with a fine-tuned MPXV metagenomic sequencing method with MPXV DNA enrichment.

Results showed significant differences when comparing depth and read count obtained using both methods. Specifically, the change in read count from the first quality control step (a typical procedure for any Illumina paired short reads) to the step where human reads are removed (Table 1, step “BMTagger”), showed a median reduction of 4±3.6% ($n=10$) for the enrichment protocol, whereas a median reduction of 94.3±3.5% ($n=2$) was determined when using the non-enrichment protocol. Furthermore, when comparing the original reads with the final quality controlled reads, the MPXV DNA enrichment approach lost around 48.26±9.93% ($n=10$) of the reads, while the non-enrichment method lost 95.25±3.45% ($n=2$). The low yield of the non-enrichment method fits with the results of other publications in the field. Additionally, the median depth when aligning the cleaned reads to the reference genome ON563414.3 in paired samples was 1500-1800 for MP01CHUAC and MP03CHUAC and 80-100 for MP01bCHUAC and MP03bCHUAC. Interestingly, the rest of samples sequenced in run C (Table 2) had a median depth of 296.5±137 ($n=8$), which indicates that the enrichment method obtained a better yield when sequencing eight samples in a single run than when sequencing just two, non-enriched, samples such as MP01bCHUAC and MP03bCHUAC.

When comparing alignment based consensus to *de novo* assemblies, the general structure was very similar (achieving a complete genome with both approaches), showing the same substitutions. However, hotspots where long and repetitive indels were detected caused problems in both methods, differing greatly in these areas. If the objective is to obtain a completely accurate and full genome, a hybrid approach should be utilized, using both short and long reads (e.g. Illumina and Oxford Nanopore Technologies). Nonetheless, characterizing these areas may not be as important for tracking the transmission of the disease. For example, Nextstrain’s pipeline for human monkeypox includes a step where masking of several regions of the genome is performed, including the first 1500 and last 7000 bp and repetitive regions of variable length.

All samples could produce good quality alignment based consensus, but the increased depth is one of the key elements to be able to trust the observed mutations. Furthermore, improving the sequencing efficiency by removing human contamination before the sequencing procedure allows for the processing of a higher number of samples in the same run, increasing the efficiency of this protocol, as can be seen in samples from run C (Tables 1 and 2).

**Table 1:** Read counts *per* quality control step. Read counts, shown for each quality control step, are calculated taking into account forward and reverse sequences separately. MP01bCHUAC and MP03bCHUAC followed a non-enrichment method, the rest of samples followed the enrichment method. Sequencing run of each sample is also shown. Original: raw reads; Illumina QC: Typical paired-end Illumina quality control; BMTagger: Human contamination removal; Kraken2: Removal of anything except MPXV

Sample	Run	Enriched	Original	Illumina QC	BMTagger	Kraken2	Lost reads (%)
MP01bCHUAC	A	No	7,755,402	5,897,356	306,422	179,086	97.69
MP03bCHUAC	A	No	5,204,934	4,020,926	406,616	374,718	92.80
MP01CHUAC	B	Yes	6,490,118	4,745,690	4,652,024	3,360,370	48.22
MP03CHUAC	B	Yes	5,702,680	4,030,170	4,002,176	2,695,288	52.74
MP05CHUAC	C	Yes	1,344,132	1,107,962	1,086,984	895,266	33.39
MP07CHUAC	C	Yes	1,376,066	1,111,552	1,096,070	878,534	36.16
MP08CHUAC	C	Yes	1,283,180	1,065,560	1,049,670	926,132	27.83
MP13CHUAC	C	Yes	1,576,732	1,149,468	1,025,064	631,872	59.93
MP15CHUAC	C	Yes	1,577,718	1,153,646	1,081,148	766,788	51.40
MP18CHUAC	C	Yes	1,679,854	1,136,220	1,053,980	921,610	45.14
MP19CHUAC	C	Yes	1,430,186	1,063,796	999,180	739,596	48.29
MP22CHUAC	C	Yes	1,670,392	1,317,902	1,207,964	817,532	51.06

**Table 2:** Alignment statistics *per* sample. Median depth and percentage of the genome with specific depths at various points is shown for each sample using the final quality controlled reads. MP01bCHUAC and MP03bCHUAC followed a non-enrichment method, the rest of samples followed the enrichment method. Sequencing run of each sample is also shown

Sample	Run	Enriched	Median depth	Depth 50x (%)	Depth 100x (%)	Depth 1000x (%)
MP01bCHUAC	A	No	102	99.77	56.85	0
MP03bCHUAC	A	No	83	95.43	39.30	2.43
MP01CHUAC	B	Yes	1,869	100	100	99.87
MP03CHUAC	B	Yes	1,576	100	100	99.78
MP05CHUAC	C	Yes	329	99.99	99.94	0
MP07CHUAC	C	Yes	400	100	99.95	0
MP08CHUAC	C	Yes	516	100	99.97	0
MP13CHUAC	C	Yes	115	99.92	79.83	0
MP15CHUAC	C	Yes	262	99.98	99.93	0
MP18CHUAC	C	Yes	460	100	99.95	0
MP19CHUAC	C	Yes	264	99.99	99.93	0
MP22CHUAC	C	Yes	185	99.95	99.90	0

Therefore, it is clear that the described methodology works well with MPXV, but the approach does have some drawbacks. Firstly, it was designed for large viruses such as MPXV and is not expected to work as well (or at all), with smaller ones, as they require higher centrifugation speed, only reachable through ultra-centrifugation. Additionally, even though paired samples showed significant differences between the MPXV DNA enrichment and non-enrichment methods, it is a low sample size for the non-enriched samples ($n=2$) compared to the enriched ones ($n=10$). Nonetheless, the results in the enriched samples are consistent in terms of how much host DNA was present.

The proposed protocol has proven to be suitable for samples with high viral load (Ct values <30 in qPCR), but not so efficient in high Ct samples. In this case, amplicon-based sequencing protocols may be more useful, as different approaches have helped to improve coverage of MPXV genomes from samples with high Ct values [15, 16]. However, some amplicon-based sequencing protocols show some limitations. For instance, the xGen Virus Amplicon NGS Panel (IDT) does not allow the sequencing of the whole viral genome, as inverted-terminal repeat (ITR) regions are not included in the amplified regions. A different strategy, based on probe-capture of viral genomes, has shown to allow whole genome sequencing of different viruses from a single sample, such as the Twist Pan Viral Panel (Twist Bioscience) or the Viral Surveillance Panel (Illumina). Nevertheless, these panels have been scarcely used for MPXV sequencing [17] and their sensitivity and specificity with this type of samples have yet to be proven. Additionally, this strategy has a significant increase in the cost per sample, so this approach might not be interesting in epidemiological surveillance.

## Conclusions

Results showed a very significant improvement in sequencing efficiency, increasing the number of reads belonging to MPXV, the depth of coverage and the trustworthiness of the consensus sequences. All in all, this methodology enables a faster and more affordable MPXV sequencing, which are relevant factors regarding infectious disease control.

## Methods

### Samples

Clinical samples were obtained from vesicular fluid swabs and conserved in UTM viral transport medium (Copan, CA, USA). Ten samples that tested positive by qRT-PCR at the Microbiology Service of the A Coruña University Hospital (HUAC) were selected for this study. The remaining fractions of samples were stored at -20$^{\circ }$C.

### DNA extraction

Viral DNA was extracted following two different protocols. In the first protocol (non-enrichment method), DNA extraction was performed using MagNA Pure Compact Nucleic Acid Isolation Kit I (Roche, Switzerland) following the manufacturer’s instructions and using 500 $\upmu$L of UTM viral transport medium as input. The second protocol (MPXV DNA enrichment) was designed to enrich samples in MPXV particles, modified from a saponin-based differential lysis method [18] followed by high g-force centrifugations in a Z 36 HK (Hermle Labortecnik, Germany) centrifuge. The addition of saponin followed by a high NaCl concentration selectively lyses human cells without affecting viral capsids. A subsequent DNase treatment of previously lysed eukaryotic cells remarkably decreases the host DNA present in the samples. Briefly, 400 $\upmu$L of samples were centrifuged at 10,000 *g* for 5 mins at 4$^{\circ }$C. Supernatant was transferred to a tube for high g-force (Labcon, CA, USA) and centrifuged at 35,000 *g* for 30 min at 4$^{\circ }$C. Pellet was resuspended in 250 $\upmu$L of PBS supplemented with saponin 2.5% and incubated at room temperature for 10 min. After the incubation, 350 $\upmu$L of water were added and incubated for 30 s, and 12 $\upmu$L of NaCl 5 M were also added. Samples were centrifuged at 35,000 *g* for 30 min at 4$^{\circ }$C, pellets were resuspended in 100 $\upmu$L of PBS and then 100 $\upmu$L of NaCl 1 M, MgCl2 100 mM and 10 $\upmu$L of HL-SAN DNase (ArticZymes, Norway Technologies) were added. Samples were incubated at 37$^{\circ }$C for 15 min with shaking at 600 rpm. Following the incubation, samples were washed twice with 800 $\upmu$L and 1 mL of PBS and centrifuged at 35,000 *g* for 30 min at 4$^{\circ }$C after each wash. Final pellet was resuspended in 100 $\upmu$L of nuclease-free water and nucleic acids were extracted using the QIAamp MinElute Virus Spin Kit (Qiagen, Germany) following the manufacturer’s instructions. DNA quantification was performed using the Qubit dsDNA HS Assay Kit (Thermo Fisher Scientific, MA, USA).

### Library generation and sequencing

DNA prep paired-end libraries (Illumina, CA, USA) were prepared using 1-5 ng of DNA extracted following the manufacturer's protocol, except for the number of PCR cycles (15). Libraries concentration was quantified using the Qubit dsDNA HS Assay Kit (Thermo Fisher Scientific, MA, USA). DNA quality and fragment size of the libraries were evaluated using the High Sensitivity D1000 Kit for TapeStation 4150 (Agilent, CA, USA). Libraries were sequenced using a MiSeq platform (Illumina, CA, USA) with paired-end sequencing, a read length of 150 nucleotides and utilizing a V2 micro cartridge (Illumina, CA, USA).

### Bioinformatic analysis

Illumina reads were first processed using BBDuk (v. 38.96) [19] to remove PhiX contamination. Clumpify (v. 38.96) [19] was used to remove duplicates and to losslessly compress the files to minify space on disk. Finally, reads were trimmed with Trimmomatic (v. 0.39) [20] for adapter removal and quality control. Human contamination was removed using BMTagger [21]. Kraken2 (v. 2.1.2) [22] was used to classify reads using the full standard database (human, bacteria, plasmid, archaea, virus, fungi and UniVec_Core), extracting read count statistics and eliminating those that did not belong to the *Orthopoxvirus* genus. Visualization of these steps was facilitated by Pavian (v. 1.2.0) [23] and KrakenTools [22]. Other measures, such as read count at each quality control step were calculated with seqkit (v. 2.1.0) [24] Read quality was assessed before and after the entire cleaning process with FastQC (v. 0.11.9) [25] and MultiQC (v. 1.11) [26].

Reads that passed all the filters were aligned to the reference genome “MPXV_USA_2022_MA001” (ON563414.3) using BWA (v. 0.7.17-r1188) [27]. Duplicates were then marked with Picard (v. 2.27.4) [28] and alignment statistics (coverage, depth, aligned reads...) were calculated with BBMap’s pileup module (v. 38.96) [19]. A consensus sequence was then produced using iVar (v. 1.3.1) [29] (parameters: “-q 20 -t 0.5 -m 10”).

The alignment based consensus method was also compared to a *de novo* assembly approach, mainly due to the possible large indels that occur in MPXV. However, because of the large inverted terminal repeats (ITRs) in MPXV, *de novo *assemblies using a short read strategy can fail to represent both of these repetitive areas at the same time. In order to solve this, random subsets of N reads are made, which create different assemblies that when merged together can create a good scaffold, which is then polished using reads. This method can also utilize different assemblers and is inspired by Tricycler’s approach [30]. Possible drawbacks include: requiring high coverage and large repetitive insertions not being resolved with only short reads.

Various assemblies were created for each sample with Unicycler (v. 0.5.0) [31] (parameters: “–linear 1”) and SPAdes (v. 3.15.4) (parameters: “–trusted-contigs $ref -k 31,51,71”) [32] using subsets of reads chosen randomly by seqtk (v. 1.3-r106) [33]. The different assemblies were then aligned to the reference MPXV genome ON563414.3 with minimap2 (v. 2.24-r1122) [34], and a consensus was made using samtools (v. 1.15) [35]. Finally, the consensus was polished with Polypolish (v. 0.5.0) [36], the reads were aligned back to the polished genome and another consensus was made with iVar (v. 1.3.1).

Nextclade (v. 2.3.0) [37] was used to denote the lineages and to quickly visualize the quality of the sequences and their mutations. An SNV comparison was also created with snipit [38]. A more in depth phylogenomic analysis was made to see the relatedness of the study’s samples to all 275 complete MPXV genomes from taxid 10244 in GenBank up to 2022-07-18 (Table S2). Sequences were aligned with Mafft (v. 7.453) [39] (parameters: “–auto”) to create a FASTA alignment, which was then transformed into PHYLIP format and used as input to RAxML (v. 8.2.12) [40] (parameters: “-m GTRCAT -T 60 -n tree -p 1 -N 1000 -p 12345 -x 12345 -f a”). The resulting tree was visualized with the Interactive Tree of Life (iTOL, v.6.5.8) [41].

## Supplementary information

**Additional file 1.** Ten samples were used in this study. Out of those, two samples, MP01 and MP03 (anonymized identifiers), were each treated with two different protocols. MP01CHUAC and MP03CHUAC samples were applied a MPXV DNA enrichment method, while MP01bCHUAC and MP03bCHUAC samples were applied a non-enrichment protocol. The rest of samples were treated with the MPXV DNA enrichment method.

**Additional file 2.** All 275 complete MPXV genomes from taxid 10244 in GenBank up to 2022-07-18 used in the phylogenomic analysis.

## Data Availability

Consensus sequences for MP01CHUAC and MP03CHUAC are available in GenBank as OP120937 and OP120938, respectively. Cleaned reads containing only reads from monkeypox for all 10 samples (12 libraries) are available under bioproject PRJNA863094. Full information available in Table S1.

## Abbreviations

ITRs: Inverted terminal repeats
SNVs: Single nucleotide variants
hMPX: Human monkeypox
MPXV: *Monkeypox virus*
